# Contemporary use and trends in percutaneous coronary intervention in Japan: an outline of the J-PCI registry

**DOI:** 10.1007/s12928-020-00669-z

**Published:** 2020-05-21

**Authors:** Mitsuaki Sawano, Kyohei Yamaji, Shun Kohsaka, Taku Inohara, Yohei Numasawa, Hirohiko Ando, Osamu Iida, Toshiro Shinke, Hideki Ishii, Tetsuya Amano

**Affiliations:** 1 Department of Cardiology, Keio University School of Medicine, Tokyo, Japan; 2 Division of Cardiology, Kokura Memorial Hospital, Kitakyushu, Japan; 3 Division of Cardiology, Vancouver General Hospital, Vancouver, BC Canada; 4 Department of Cardiology, Japanese Red Cross Ashikaga Hospital, Ashikaga, Japan; 5 Department of Cardiology, Aichi Medical University, Nagakute, Japan; 6 Department of Cardiology, Cardiovascular Center, Kansai Rosai Hospital, Amagasaki, Japan; 7 Department of Cardiology, Showa University School of Medicine, Tokyo, Japan; 8 Department of Cardiology, Fujita Health University Bantane Hospital, Nagoya, Japan

**Keywords:** CVIT, J-PCI registry, Registry comittee, NCD

## Abstract

Cardiovascular interventions have achieved a level of excellence, with many outstanding advanced techniques and results. The mission of the Japanese Association of Cardiovascular Intervention and Therapeutics (CVIT) is to further our understanding of cardiovascular intervention and establish its procedural safety. [1] The Japanese Percutaneous Coronary Intervention (J-PCI) registry was established and sponsored by CVIT, and aims to provide basic statistics on the performance of percutaneous coronary interventions (PCI) in Japan. Today, the database has grown to become one of the largest healthcare procedural database with more than 200,000 cases registered annually from approximately 900 institutions in Japan representing over 90% of all PCI hospitals in the nation. Importantly, case registrations in the J-PCI registry are essential for coronary interventionalist and educating hospital certification. The present manuscript aimed to summarize the history of the J-PCI registry and outline the definitions of various items.

**Electronic supplementary material:**

The online version of this article (10.1007/s12928-020-00669-z) contains supplementary material, which is available to authorized users.

## Background and aims

The J-PCI registry was launched in 2007 with the University Hospital Medical Information Network (UMIN) as a case registration platform for PCI procedures performed within the country (directed by Shinsuke Nanto of Osaka University; 2,925 cases registered in the first year). The main objective of PCI case registration within the J-PCI registry was to establish PCI as a safe treatment for all Japanese patients with coronary artery disease. In the following year, the primary objective of the J-PCI was defined as follows:

1. To collect reliable data and accurately describe the entirety of PCIs performed within country.
2. Seek for unmet needs regarding PCI, and further conduct in-depth analysis to find potential solutions.
3. Perform consecutive case registration as a prerequisite for institution certification, and audit data regularly to ensure its accuracy.

**Fig. 1:**
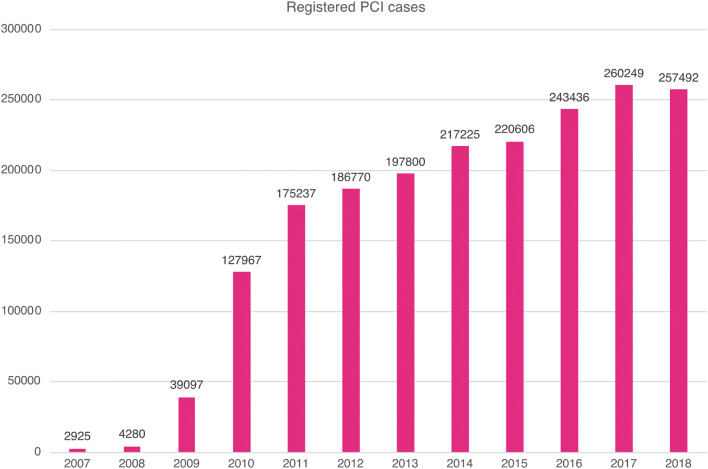
Annual numbers of cases registered in the J-PCI since its launch in 2007

The annual number of cases registered within the registry exceeded 100,000 in 2010 and rapidly increased to over 200,000 in 2013 (Fig. 1). During this time, the J-PCI worked in alliance with the Japan Cardiovascular Surgery Database (JCVSD) [2–6] (directed by Shinichi Takamoto of The University of Tokyo), in which coronary artery bypass and all cardiothoracic surgeries are registered. The J-PCI registration data platform was transferred from UMIN to the National Clinical Database (NCD) [7, 8] and a working group was formed within the Japanese Circulation Society.

The subsequent utilization of the NCD system has gradually resulted in coordination between, and joint management of, the J-PCI registry data and the coronary interventionalist/institution certification system. In 2015, three key variables were added on to the J-PCI case report form to enrich case characteristics: (1) medications utilized prior to catheterization, (2) presence of myocardial ischemia proven by various cardiac imaging prior to catheterization for non-emergent cases, and (3) door-to-balloon time for ST-elevation myocardial infarction (STEMI) cases. In addition, academic research proposals are being accepted each year owing to the registry’s ability to collect clean and reliable data (research proposals submitted every September, with 3–4 proposals accepted as research projects every year). Titles and summary of previously published peer-reviewed articles from the J-PCI registry are shown in Table 1.

**Table 1:** Titles and brief summary of published articles from the J-PCI registry

No.	Title	Authors	Citation	Summary
1	Incidence and Determinants of Complications in Rotational Atherectomy: Insights From the National Clinical Data (J-PCI Registry) [20]	Sakakura K, Inohara T, Kohsaka S, Amano T, Uemura S, Ishii H, Kadota K, Nakamura M, Funayama H, Fujita H, Momomura SI	Circ Cardiovasc Interv. 2016 Nov;9 (11). pii: e004278	The reported incidence of rotational atherectomy procedure-related complication rate was 1.3%, with each component ranging between 0.2 and 0.6%. Age, impaired kidney function, and previous myocardial infarction, emergent procedures, number of diseased vessels, and low institutional volume of radial access intervention were associated with higher complication rates
2	Relation of ST-Segment Elevation Myocardial Infarction to Daily Ambient Temperature and Air Pollutant Levels in a Japanese Nationwide Percutaneous Coronary Intervention Registry [9]	Yamaji K, Kohsaka S, Morimoto T, Fujii K, Amano T, Uemura S, Akasaka T, Kadota K, Nakamura M, Kimura T; J-PCI Registry Investigators	Am J Cardiol. 2017 Mar 15;119(6):872–880	Absolute value and relative change in the ambient temperature were associated with the occurrence of STEMI; the associations with the air pollutant levels were less clear after adjustment for these meteorologic variables in this nationwide database
3	Impact of Institutional and Operator Volume on Short-Term Outcomes of Percutaneous Coronary Intervention: A Report From the Japanese Nationwide Registry [10]	Inohara T, Kohsaka S, Yamaji K, Amano T, Fujii K, Oda H, Uemura S, Kadota K, Miyata H, Nakamura M; J-PCI Registry Investigators	JACC Cardiovasc Interv. 2017 May 8;10(9):918–927	In contemporary Japanese PCI practice, lower institutional volume (< 150 PCIs/year) was related inversely to in-hospital outcomes, but the association of annual operator volume with outcomes was less clear
4	Comparison of Outcomes of Women Versus Men With Non-ST-elevation Acute Coronary Syndromes Undergoing Percutaneous Coronary Intervention (from the Japanese Nationwide Registry) [21]	Numasawa Y, Inohara T, Ishii H, Kuno T, Kodaira M, Kohsaka S, Fujii K, Uemura S, Amano T, Kadota K, Nakamura M	Am J Cardiol. 2017;119(6):826–831	In patients with non-ST-elevation acute coronary syndrome who underwent PCI, women were at greater risk than men for in-hospital complications, especially in bleeding complications
5	Comparison of Outcomes After Percutaneous Coronary Intervention in Elderly Patients, Including 10628 Nonagenarians: Insights From a Japanese Nationwide Registry (J-PCI Registry) [11]	Numasawa Y, Inohara T, Ishii H, Yamaji K, Kohsaka S, Sawano M, Kodaira M, Uemura S, Kadota K, Amano T, Nakamura M; J-PCI Registry Investigators	J Am Heart Assoc. 2019 Mar 5;8(5):e011183	Older patients, especially nonagenarians [10 628 patients (1.9%) of all PCI patients}, carried a greater risk of in-hospital death and bleeding compared with younger patients after PCI. Transradial intervention might contribute to risk reduction for periprocedural complications in elderly patients undergoing PCI
6	In-Hospital Outcomes After Percutaneous Coronary Intervention for Acute Coronary Syndrome With Cardiogenic Shock (from a Japanese Nationwide Registry [J-PCI Registry]) [12]	Kubo S, Yamaji K, Inohara T, Kohsaka S, Tanaka H, Ishii H, Uemura S, Amano T, Nakamura M, Kadota K	Am J Cardiol. 2019 May 15;123 (10):1595-1601	In-hospital mortality was 13.2% in ACS patients with cardiogenic shock who underwent contemporary PCI. Lower institutional PCI volumes, and concurrent bleeding were associated with higher in-hospital mortality
7	Risk stratification model for in-hospital death in patients undergoing percutaneous coronary intervention: a nationwide retrospective cohort study in Japan [13]	Inohara T, Kohsaka S, Yamaji K, Ishii H, Amano T, Uemura S, Kadota K, Kumamaru H, Miyata H, Nakamura M	BMJ Open. 2019 May 22;9(5):e026683	We developed and validated a risk model predicting in-hospital mortality in a broad spectrum of Japanese patients after PCI. The risk model performed well in the entire validation cohort and among prespecified subgroups with good calibration, although both models underestimated the risk of mortality in high-risk patients with the elective procedure
8	An overview of percutaneous coronary intervention in dialysis patients: Insights from a Japanese nationwide registry [14]	Numasawa Y, Inohara T, Ishii H, Yamaji K, Hirano K, Kohsaka S, Sawano M, Kuno T, Kodaira M, Uemura S, Kadota K, Amano T, Nakamura M; J-PCI Registry Investigators	Catheter Cardiovasc Interv. 2019 Jul 1;94 [1]:E1–E8	PCI was widely performed for dialysis patients with either ACS or non-ACS in Japan. Dialysis patients had a greater risk of adverse outcomes compared to nondialysis patients after PCI
9	Post-interventional adverse event risk by vascular access site among patients with acute coronary syndrome in Japan: observational analysis with a national registry J-PCI database [15]	Fujii T, Ikari Y, Hashimoto H, Kadota K, Amano T, Uemura S, Takashima H, Nakamura M; J-PCI Investigators	Cardiovasc Interv Ther. 2019 Oct;34 [4]:297–304	Radial access was related to a significantly lower risk for access site bleeding compared with femoral access, even without strong antithrombotic drugs for ACS in Japan, and may also relate to lower risk for a wider set of post-treatment adverse events
10	Impact of Reduced-Dose Prasugrel vs. Standard-Dose Clopidogrel on In-Hospital Outcomes of Percutaneous Coronary Intervention in 62,737 Patients with Acute Coronary Syndromes: A Nationwide Registry Study in Japan [16]	Akita K, Inohara T, Yamaji K, Kohsaka S, Numasawa Y, Ishii H, Amano T, Kadota K, Nakamura M, Maekawa Y	Eur Heart J Cardiovasc Pharmacother. 2019 Oct 8	In Japanese ACS patients undergoing PCI, the risk of bleeding was higher when using reduced-dose prasugrel than when using standard-dose clopidogrel, but there is no significant difference in in-hospital mortality and incidence of stent thrombosis between the two antiplatelet regimens
11	Diabetes mellitus and other cardiovascular risk factors in lower-extremity peripheral artery disease versus coronary artery disease: an analysis of 1,121,359 cases from the nationwide databases [17]	Takahara M, Iida O, Kohsaka S, Soga Y, Fujihara M, Shinke T, Amano T, Ikari Y; J-EVT and J-PCI investigators	Cardiovasc Diabetol. 2019 Nov 15;18 [1]:155	Patient profiles were not identical but rather considerably different between clinically significant lower-extremity peripheral artery disease and coronary artery disease patients warranting revascularization. Of note, the prevalence of diabetes mellitus and end-stage renal disease was 1.96- and 6.39-times higher in LE-PAD patients than in CAD patients
12	Presentation Pattern of Lower Extremity Endovascular Intervention versus Percutaneous Coronary Intervention [18]	Takahara M, Iida O, Kohsaka S, Soga Y, Fujihara M, Shinke T, Amano T, Ikari Y; J-EVT and J-PCI investigators	J Atheroscler Thromb. 2019 Nov 21	Compared with acute coronary syndrome patients, critical limb ischemia demonstrated a larger peak-to-trough ratio of seasonality (1.75 versus 1.21; *P*<0.001), and a later peak appearance. These distinct features were observed in a diabetic population and a non-diabetic population
13	Incidence and In-Hospital Outcomes of Patients Presenting With Stent Thrombosis [19]	Ohno Y, Yamaji K, Kohsaka S, Inohara T, Amano T, Ishii H, Kadota K, Nakamura M, Nakazawa G, Yoshimachi F, Ikari Y	Am J Cardiol. 2019 Dec 9. pii: S0002-9149(19)31361-X	Despite younger age, patients with ST had significantly higher incidence of in-hospital mortality and cardiovascular complications, including recurrent ST, compared with those without

## Overall design and concept of the registry

The J-PCI registry is a cross-sectional nationwide PCI registry linked with coronary interventionalist and training hospital certification within CVIT, a professional coronary interventionalist academic society. All data on PCIs performed by CVIT members are entered into a unique electronic data capturing (EDC) system provided by the NCD. With regard to clinically relevant items, efforts are made to gather a variety of items based on the reproducibility and feasibility of data input. Designated data entry operators and data managers per institution can access the EDC website to register and edit case data. Credibility of the data are maintained systematically through a double-check system by enabling one person to enter data and another person to recheck and finalize the entered data. Once the data are finalized, the data cannot be accessed from the individual institution personnel. Nonetheless, queries are gathered and answered at the CVIT headquarters or the NCD to enable interactions with the EDC users and administrators to continuously improve our system.

Data manager meetings are held every year during the annual CVIT congress as well as at satellite congress held to (1) notify addition/exclusions in collected variables and definitions, and (2) answer or debate on controversial issues provided by individual data entry operators and managers. Data audit via random site visiting (10–20 hospitals/year) is performed each year to identify root causes and correct data errors. As of 2019, the J-PCI registry operations are managed by the CVIT registry committee (current chair: Tetsuya Amano, Aichi Medical University) established in summer 2018. The summary data are presented every year at the annual CVIT congress and presented on the CVIT website (http://www.cvit.jp/registry/annual-report.html). In addition to the J-PCI registry, the CVIT also manages the J-EVT (EndoVascular Treatment)/SHD (Structural heart disease) registry.

## Clinical variables collected in the J-PCI registry

### Preprocedural patient characteristics

The focus of the registry is to depict the characteristics of PCI with the least but sufficient number of variables per case in respect to structural, procedural, and outcome for quality measurement and standardization. All items were selected based on their relation with periprocedural outcomes including in-hospital death (e.g. out-of-hospital cardiac arrest, cardiogenic shock, presence of diabetes, hypertension, dyslipidemia, smoking, chronic lung disease, peripheral vascular disease, chronic kidney disease and hemodialysis) (Definitions shown in Table 2). In addition, pretreatment medication status regarding antiplatelet drugs (aspirin, clopidogrel, prasugrel, ticagrelor, etc.) and anticoagulants (warfarin, dabigatran, rivaroxaban, apixaban, edoxaban, etc.) as well as preprocedural hemoglobin and creatinine levels are registered regarding their impact on bleeding events. For patients with stable coronary artery disease (CAD), performance of preprocedural noninvasive cardiac testing such as coronary computed tomography (CT) angiography, treadmill exercise testing, single-photon emission computed tomography (SPECT), stress transthoracic echocardiogram (TTE), stress magnetic resonance imaging (MRI) as well as invasive physiological indices measurement devices are registered.

**Table 2:** Definitions of key baseline variables

Diabetes	At least one of the following criteria is met: (a) Fasting blood glucose ≥ 126 mg/dL (b) Random blood glucose ≥ 200 mg/dL (c) HbA1c ≥ 6.5 (as per Japanese formula) (d) 2-h 75 g OGTT blood glucose ≥ 200 mg/dL (e) Treatment with oral antidiabetic agents, insulin, or incretin medication
Hypertension	At least one of the following criteria should be met based on the Japanese Society of Hypertension 2009 guideline: (a) Systolic blood pressure ≥ 140 mmHg (b) Diastolic blood pressure ≥ 90 mmHg (c) Undergoing treatment with antihypertensive agents
Dyslipidemia	Any of the following are met based on the Japan Atherosclerosis Society (JAS) Guidelines for Prevention of Atherosclerotic Cardiovascular Diseases 2012 [20] LDL cholesterol ≥ 140 mg/gL HDL cholesterol < 40 mg/dL Triglycerides ≥ 150 mg/dL LDL cholesterol is calculated using the Friedewald formula (TC–HDL-C–TG/5) (when TG < 400 mg/dL). When TG is ≥ 400 mg/dL or using postprandial blood, non-HDL-C (TC–HDL-C) should be used *“Fasting” is defined as taking no food for over 10–12 h
Smoking	All patients with a history of smoking within the past year
Chronic kidney disease	At least one of the following criteria should be met (Japanese Society of Nephrology CKD Treatment Guidelines 2009): (a) Proteinuria (b) Serum creatinine ≥ 1.3 mg/dL (c) eGFR ≤ 60 ml/min/1.73 m^2^ (eGFR = 194 × age − 0.23 × Cre − 0.1154 [women × 0.742])
Maintenance dialysis	Undergoing hemodialysis or peritoneal dialysis

*HbA1c*, Hemoglobin A1c, *OGTT* Oral glucose tolerance test, *LDL* Low-density lipoprotein cholesterol, *HDL* High-density lipoprotein, *TG* Triglyceride, *CKD* Chronic kidney disease, *eGFR* Estimated glomerular filtration rate, *Cre* Creatinine

### Preprocedural coronary lesion characteristics

Diagnosis resulting in hospitalization is first classified based on the presence or absence of chest pain symptoms within the previous month and further subdivided into the following categories as shown in Table 3.

**Table 3:** Definitions of Categories upon Clinical Presentation

Stable angina	Angina with stable symptoms in the past month, with no symptom attacks at rest (symptoms only elicited during high exertion, with no changes in frequency or intensity in the past month)
Unstable angina	At least one of the following is met: 1) New-onset angina: Angina, which manifested within the past month 2) Increasing angina: angina that worsened within the past month 3) Resting angina: persistent angina at rest or angina that markedly restricts daily life (symptoms triggered by walking tens of meters or one flight of stairs) 4) Postinfarction angina: persistent angina within 1 month following a myocardial infarction event with the involvement of elevated ST segments on ECG or cardiac biomarkers; if they are, the angina is defined as STEMI or NSTEMI, respectively
Acute myocardial infarction	Persistent myocardial ischemia symptoms accompanied by elevated cardiac markers. Elevated cardiac biomarkers refers to elevated creatine kinase (CK) or CK-MB levels [two-folds higher than the normal values] or elevated troponin levels [≥ 99th percentile] Acute myocardial infarctions are classified as STEMI or NSTEMI as described below: 1) ST-elevation myocardial infarction (STEMI): ST elevation on two or more contiguous leads (≥ 0.2 mV in a precordial lead at the J point or ≥ 0.1 mV in a limb lead), new left bundle branch block, or posterior myocardial infarction on a 12-lead ECG. 2) Non-ST-elevation myocardial infarction (NSTEMI): ECG changes either do not qualify as ST elevation or are not present at all
Stent thrombosis	Definite stent thrombosis as defined by the Academic Research Consortium (ARC) (described below). 1. Angiographic confirmation of stent thrombosis The presence of a thrombus that originates from the stent or the segment 5 mm proximal or distal to the stent, and the presence of at least one of the following criteria within a 48-h period: 1) Acute onset of ischemic symptoms at rest 2) New ischemic ECG changes indicative of acute ischemia 3) Typical rise and fall in cardiac biomarkers 2. Pathological confirmation of stent thrombosis Evidence of recent thrombus within the stent at autopsy or by examination of tissue retrieved following thrombectomy
Previous myocardial infarction	At least one of the following is met: 1) New abnormal Q wave on an ECG in two or more contiguous leads without evident chest symptoms 2) Confirmation of segmental non-viable myocardium in imaging tests without evident chest symptoms
Silent ischemic myocardial infarction	Confirmation of ischemia on a stress ECG or imaging tests (SPECT, stress TTE, stress MRI, etc.) without evident chest symptoms in the past month

*ECG* Electrocardiogram, *STEMI* ST-Elevation Myocardial Infarction, *NSTEMI* Non-ST-Elevation Myocardial Infarction, *CK-MB* Creatine kinase-MB, *SPECT* Single-photon emission computed tomography, *TTE* Transthoracic echocardiogram, *MRI* Magnetic resonance imaging

### Procedural characteristics

As of 2019, the following items can be selected as treatment device per lesion: coronary balloon, drug-eluting balloon, bare-metal stent, drug-eluting stent, bioresorbable vascular scaffold, rotational atherectomy device, directional coronary atherectomy device, thrombus aspiration, distal protection, the use of mechanical circulatory support devices such as the Impella®, procedural success/failure and access site. With regard to coronary artery information, this section also includes the number of stents placed. Successful PCI is defined as ≤ 10% of the remaining stenosis following stent placement. Door to balloon time is registered if in the case of STEMI, and total procedure time and total contrast dose are registered for each PCI.

### In-hospital outcomes

In-hospital outcomes include the following variables within the J-PCI registry: mortality, postprocedural myocardial infarction, cardiac tamponade, acute heart failure/cardiogenic shock, stent thrombosis, access/nonaccess site bleeding events requiring red blood cell transfusion and emergency surgery. From January 2019, a minor revision in the definition of in-hospital mortality has been changed from 30-day mortality to all mortality during hospitalization with the addition of cause of death classified to cardiac, noncardiac or unknown cause. Validation studies on in-hospital outcomes have been rather difficult to conduct due to the nature of the J-PCI registry: auditing has not been conducted in a complete manner. However, evaluation has been conducted in the past to verify the volume outcomes of PCI. In this analysis, in-hospital outcomes were stable for institutions which performed ≥ 200 PCIs per year, but no clear cutoff point was observed for annual operator volume [10.]

## Ancillary studies derived from the main J-PCI Registry

### One-year follow-up registry (J-PCI OUTCOME Registry)

In 2017, a milestone project in collaboration with the Japanese Circulation Society and funded by the Japan Agency for Medical Research and Development (AMED), a 1-year long-term nationwide cohort was launched based on the J-PCI registry 2017 data. Out of more than 900 hospitals in the J-PCI, 179 hospitals volunteered to participate in this cohort project. The study protocol was approved by a third-party ethics committee at Osaka University as the central institutional review board as well as the local institutional review board of each site. The 2017 cohort data were entered from August, 2018 to March, 2019. Over 49,014 patients were followed with 16,129 thousand-person days with a follow-up rate of 79.3% for the initial January 1st to December 31st, 2017 cohort. The cohort is expected to continue until the J-PCI 2018 Registry outcome data entry (expected to be finished by Spring, 2020. The J-PCI OUTCOME cohort is the first of its kind to capture incident fatal and nonfatal events after initial PCI including planned revascularization at a nationwide scale in Japan. One-year mortality including mode of death, nonfatal events (up to a maximum of 3 episodes) necessitating hospitalization for acute coronary syndromes, stroke, bleeding, acute heart failure and planned revascularization (up to a maximum of 5 episodes) are registered in the cohort, Endpoint were defined according to the 2017 Cardiovascular and Stroke Endpoint Definitions for Clinical Trials definitions.

### Hospital-based feedback system on quality metrics

In January 2018, the CVIT launched its first hospital-based feedback system via the NCD website for data managers per site. The feedback system is consisted of 7 components: proportion of ACS patients, proportion of emergent or urgent PCI, door-to-balloon time for STEMI patients, proportion of patients with preprocedural antiplatelet agent, proportion of transradial PCI, proportion of patients that underwent preprocedural ischemia evaluation prior to elective PCI and proportion of patients undergoing distal coronary lesion PCI. These components were determined after discussion with the current registry members by accounting its importance towards PCI quality improvement and data collection feasibility. The feedback system aims to standardize the quality of PCI within the nation and the impact of the system is scheduled to be analyzed by comparing facilities that visited the feedback website vs those who did not in the J-PCI registry after the year of 2018. The launch of the website was notified to data managers of each participating hospital via email without any additional incentives or penalties set a priori in 2018. In 2019, all data managers wishing to renew their CVIT certification are obliged to print out the reports on the NCD website and comment on achievable goals in attempt to improve the quality of PCI in their institution. While these efforts are still in their infancy, they are a significant step for CVIT as an academic society that will develop into a response to the ever-growing social demand for equitable distribution of medical resources.

## Conclusion

The main mission of the J-PCI is to improve the quality of patient care and establish procedural safety by providing information to the CVIT community members. The J-PCI data provide broad and unique perspectives into the care and outcomes of cardiovascular diseases in Japan. Through the contributions of the participating hospitals and physicians, this nationwide registry will continue to provide standardized data to advance the care of cardiovascular disease.

## Electronic supplementary material

Below is the link to the electronic supplementary material.

Supplementary file1 (DOCX 45 kb)

## Abbreviations

ECG: Electrocardiogram
STEMI: ST-elevation myocardial infarction
NSTEMI: Non-ST-elevation myocardial infarction
CK-MB: Creatine kinase-MB
SPECT: Single-photon emission computed tomography
TTE: Transthoracic echocardiogram
MRI: Magnetic resonance imaging
HbA1c: Hemoglobin A1C
OGTT: Oral glucose tolerance test
LDL: Low-density lipoprotein
HDL: High-density lipoprotein
TG: Triglyceride
CKD: Chronic kidney disease
eGFR: Estimated glomerular filtration rate
Cre: Creatinine
