# Supplementary material for: Contemporary use and trends in percutaneous coronary intervention in Japan: an outline of the J-PCI registry
Source: Cardiovasc Interv Ther. 2020 May 21;35(3):218–26. doi: 10.1007/s12928-020-00669-z (PMC7295726; doi:10.1007/s12928-020-00669-z)
Supplement: Supplementary file 1 — Supplementary file1 (DOCX 45 kb) [file 12928_2020_669_MOESM1_ESM.docx]

National Clinical Database:Case Report Form Guidance for completing

J-PCI

Caution

This Case Report Form (hereafter referred to as CRFs) is a supplementary document for enrolling patients in the National Clinical Database (hereafter referred to as NCDs). See this CRF for definitions of each item.

This CRF is only a supplementary document, so the final registration of cases should be performed via the Web site.

If this CRF is sent to NCD, it cannot be regarded as a case registration. Please be advised. This CRF should be used in the following situations.

Rules for completing

The entry rules are as follows: The rule is the same on the website for the icons of ● and ●...

○: This is an alternative item. Choose only one of the appropriate items.

□: This is a multiple choice item. Which of the following items should be selected? Underlined is the column for entering text.

Item names are shaded in gray, which is a mandatory item.

Contact Us

If you have any unknown questions or questions, please contact the NCD Web site via the Contact Form.

(URL http://www.ncd.or.jp/contact)

Please also refer to the FAQ prior to your inquiry. (URL http://www.ncd.or.jp/faq)

※ I do not accept the telephone inquiry because I manage it centrally by e-mail. Please appreciate your graduation. Depending on the contents of your inquiry, it may take a long time to answer.

| Diagnosis leading to hospitalization ① [Classification of patients with symptoms within 1 month] | |
| --- | --- |
| Stable angina | Angina pectoris with stable symptoms for the last month and no symptoms at rest (= induction of symptoms has not changed in frequency or severity for 1 month only on severe exertion). )。 |
| Unstable angina | One of the following is true.  ① NewOnsetAngina: Angina pectoris in the last month  ② IncreasingAngina: Angina pectoris progressed in the last month  ③ RestingAngina: Persistent angina at rest or severely limited daily living Angina (Symptoms develop in several meters walking, more than one step on stairs, etc.)  ④ Post-infarct angina: Angina pectoris that persists within 1 month of an MI event.  But without elevation of ST-or cardiac markers (or STEMI, if present)  You have NSTEMI. )。 |
| Acute myocardial infarction | Persistent myocardial ischemia with elevated cardiac markers. Elevated cardiac markers are CK or CK-MB > 2 times normal or troponin > 99th percentile. It is classified into the following STEMI and NSTEMI. 1) ST-segment elevation myocardial infarction (STEMI): ST-segment elevation (> 0.2mV in thoracic leads and > 0.1mV in limb leads at the J point), new left bundle branch block, or pure posterior infarction in two or more leads on a 12-lead ECG.  2) Non-ST-segment elevation myocardial infarction (NSTEMI): ECG changes do not belong to the ST-segment elevation type or are absent. |
| Stent thrombosis | Definite under definition for Academic Research Consortium (see below).... This section can be selected in duplicate with the above sections.  Definition of stent thrombosis ARC   1. Angiographic confirmation of stent thrombosis   Thrombi originating in the stent or at the 5mm end of the stent and meeting at least one of the following criteria within 48 hours.   - 1. Acute onset of resting ischemic symptoms   2. New Ischemic Electrocardiographic Changes Suggesting Acute Ischemia   3. Typical Elevation and Lowering of Myocardial Markers  1. Confirmation of stent thrombosis by pathology   Histological findings of subacute in-stent thrombosis after necropsy or thrombectomy. |

| Diagnosis leading to hospitalization ② [Classification of patients without specific symptoms within 1 month] | |
| --- | --- |
| Old myocardial infarction | One of the following is true.   1. No obvious chest symptoms within the last month and new abnormalities on ECG   Q waves are present in two or more adjacent leads.   1. If no obvious chest symptoms have been present in the last month and imaging demonstrates segmental nonviable myocardium (infarction: thinning and lack of contractility). |
| Painless ischemic myocardial infarction | If no obvious chest symptoms have been present in the last month and segmental ischemia has been documented on stress ECG or imaging studies (e.g., myocardial scintigraphy, echocardiography, MRI). |

| Intercurrent illness | |
| --- | --- |
| Diabetes mellitus | One of the following is true.  (a) Fasting dose of 126mg/dl or more  (1) Occasional blood glucose 200mg/dl  (c) HbA1c6. 1 or more (equivalent to 6.5% in foreign countries according to the Japanese formula)  (e) The 2-hour blood glucose level of 75gOGTT is 200mg/dl or more.  (o) Treatment with oral hypoglycemic agents, insulin, and incretin preparations |
| Hypertension | Japanese Society of Hypertension Guidelines 2009: One of the following is met.  (a) SBP140mmHg or higher  (1) DBP90mmHg or higher  (c) Treatment with antihypertensive drugs |
| Dyslipidaemia | Dyslipidemia: Any diagnostic criterion for screening (fasting blood sampling*) was fulfilled (although borderline high LDL cholesterolaemia was excluded from the conditions). )。   \| LDL cholesterol \| 140 mg/dL or more \| High LDL cholesterolaemia \| \| --- \| --- \| --- \| \| HDL cholesterol \| Less than 40 mg/dL \| Hypo-HDL-cholesterolemia \| \| Triglyceride \| 150 mg/dL or more \| Hypertriglyceridemia \|   LDL-cholesterol is calculated by the equation Friedewald(TC-HDL-C-TG/5 (TG,TG-cholesterol,LDL-cholesterol  Less than 400 mg/dL).  Non HDL-C(TC-HDL-C) should be used if TGs are ?400mg/dL or postprandial blood sampling, and the standards should be LDL-C + 30 mg/dL.  ※ Fasting for 10-12 hours or longer is considered "fasting." However, water and tea that are calorie-free can be consumed.  Source: Japanese Atherosclerosis Society. Chapter 3 Diagnostic criteria for dyslipidemia. Guidelines for the Prevention of Atherosclerotic Diseases 2012. 2012; 33-36. |
| Cigarette smoking | Any history of smoking within the past year. |
| Renal failure | Japanese Society of Nephrology CKD Practice Guide 2009: One of the following is met.  (a) Presence of proteinuria  (1) S-Cr1 ?3mg/dl  (c) eGFR60ml/min/1. 73m2 or less (eGFR = 194 × Age-0. 23 × Cre-0. 1154 [woman × 0.742]) |
| Maintenance dialysis | Artificial dialysis and peritoneal dialysis are being performed. |

| Complication of the procedure | |
| --- | --- |
| Myocardial infarction due to PCI procedures | Integrated Q-wave infarction and non-Q-wave infarction. See Universal Definition of Myocardial Infarction: Myocardial infarction associated with PCI is defined as an increase of at least 5-fold the upper limit of normal for cTn.(abridged author abst.) |
| Cardiac tamponade | If pericardial drainage is required due to accumulation of pericardial effusion with new hemodynamic deterioration after PCI. |
| Heart failure or shock (cardiogenic shock) requiring assisted circulation or inotropic drugs | Hypotensive state (systolic blood pressure < 90mmHg or mean blood pressure) that does not improve with fluid therapy  Less than 60 mmHg). For long-term hospitalized patients, events beyond 30 days after PCI are not included. |
| Stent thrombosis (nosocomial onset) | Definitive definite of ARCs.  Definition of stent thrombosis ARC   1. Angiographic confirmation of stent thrombosis   Thrombi originating in the stent or at the 5mm end of the stent and meeting at least one of the following criteria within 48 hours.   - 1. Acute onset of resting ischemic symptoms   2. New Ischemic Electrocardiographic Changes Suggesting Acute Ischemia   3. Typical Elevation and Lowering of Myocardial Markers  1. Confirmation of stent thrombosis by pathology   Histological findings of subacute in-stent thrombosis after necropsy or thrombectomy. |
| Emergency surgery | Unscheduled surgery performed during the same hospital stay to avoid exacerbation of risk  Surgery. |
| Hemorrhagic complications requiring transfusion (access sites) | Blood transfusion is required due to intraoperative or postoperative bleeding, and bleeding or type around the puncture site such as femoral artery or radial artery |
| Hemorrhagic complications (non-access sites) requiring transfusion | Blood transfusion is required due to intraoperative or postoperative bleeding, and bleeding from a site unrelated to the puncture site such as the femoral artery or the radial artery or the like or the type of bleeding |

| Hospital mortality | |
| --- | --- |
| Hospital mortality | Death in hospital, any number of days after PCI. |
| Causa mortis | Cardiac death includes death from acute coronary syndrome (ACS), sudden cardiac death, death from heart failure, death from cerebral infarction, death from cardiac procedures or surgery, death from major hemorrhage from the heart, and death caused by the heart as a major cause (*). All deaths are considered cardiac unless there is a clear cause of noncardiac death.  (Circulation. 2018;137:961-972)  ※ Major factors are those that underlie the sequence of events that ultimately lead to death. For example, if ACS causes a fatal arrhythmia, the primary cause is ACS.  Procedure-related death is the death of a cardiac death that is directly attributable to complications from PCI or another invasive procedure. |

**National Clinical Database Case Report Form:J-PCI**

# Patient information

| Date of initial registration. | Year Month Day | Automatic entry on the case registration system. |
| --- | --- | --- |
| Hospital management code  (Site Patient Number) | ○ Hospital Administration Codes Not Completed by Facility Policies  , | When the hospital management code is written, it is decided by the medical care department of each facility and written. Both half-size letters and half-size numbers can be used. |
| Patient sex | 〇 Male ● Female | |
| Patient's date of birth | Year Month Day | Completion is mandatory until the month. |

**Surgery/Intervention Information Preoperative Information**

| History of PCI | ○ Presence ○ No ○ Unknown | CABG history | ○ Presence ○ No ○ Unknown |
| --- | --- | --- | --- |
| History of myocardial infarction | ○ Presence ○ No ○ Unknown | | |
| History of heart failure | ○ Presence ○ No ○ Unknown | Cases diagnosed as cardiac insufficiency. However, patients with left ventricular dysfunction without symptoms are excluded. | |
| Cardiopulmonary arrest within 24 hours | ○ Presence ○ No ○ Unknown | Cardiac arrest (asystole), ventricular fibrillation (VF), and ventricular tachycardia without pulse (pulseless VT) requiring cardiopulmonary resuscitation within 24 hours prior to PCI. | |
| Cardiogenic shock within 24 h | ○ Presence ○ No ○ Unknown | Subjects who were clinically diagnosed with circulatory failure (any of the following) within 24 hours prior to PCI.  ・ Systolic blood pressure < 80 mm Hg and/or cardiac index < 1.8 l/min/m2 despite maximal treatment  ・ Intravenous administration of cardiotonic or other drugs and IABP supportive measures are required to maintain systolic blood pressure >80mmHg and cardiac index > 1.8 l/min/m∞2. | |
| Acute heart failure within 24 h | ○ Presence ○ No ○ Unknown | Subjects who are diagnosed with acute heart failure within 24 hours prior to PCI and have symptoms even at rest or on mild exertion  (equivalent to NYHA IV). Acute heart failure includes the following symptoms and signs.  ・ Dyspnea (caused by heart disease) during light exertion  ・ Orthopnea (caused by heart disease)  ・ Fluid retention (due to heart disease)  ・ Moist rale  ・ Distention of neck veins  ・ Pulmonary edema on chest x-ray | |
| Presence or absence of symptoms within 1 month | ○ Presence or Absence | | |
| Diagnosis of PCI  (Classification of patients with symptoms within 1 month)  ※ Enter if "yes" is selected in [Presence or absence of symptoms within 1 month]. | ○ Stable Angina ● Unstable Angina  ○ Acute myocardial infarction (∞ non-ST segment elevation type)  ○ ST elevation ● Unknown  Stent thrombosis | For the selection of diagnosis, see "Diagnosis leading to hospital admission ① [Classification of patients with particular symptoms within 1 month]" in the J-PCI items defines.  Only "stent thrombosis" can be selected in duplicate with other items.  If "acute myocardial infarction" is selected, selection of the lower choice (non-ST-elevation type, ST-elevation type, unknown) is mandatory. | |
| Diagnosis of PCI  (Classification of patients without any symptoms within 1 month)  ※ Enter if "None" is selected in [Presence or absence of symptoms within 1 month]. | ○ Old myocardial infarction  ○ Painless ischemic heart disease  ○ Staged PCI  ○ Other [ ] | See Definitions of J-PCI Items "Diagnosed as having been hospitalized this time ② [Classification of patients without any symptoms within 1 month]." | |
| Intercurrent illness | ● Diabetes ● Hypertension ● Dyslipidemia  ● Tobacco use ● Renal failure ● Maintenance dialysis  ● Chronic lung disease ● Peripheral vascular disorder  ○ No concurrent disease | See J-PCI Cover, Concomitant Diseases. Multiple responses possible. | |
| Preoperative imaging and stress testing  ※ If the four items of "stable angina," "old myocardial infarction," "indolent ischemic heart disease," and "others" are selected in the [Diagnosis of PCI this time]. | ○ Presence or Absence | Presence or absence of coronary artery autopsy or ischemic assessment procedures performed within 3 months of PCI | |

| Details of the examination  ※ Write if "Yes" is selected in [Perform preoperative imaging/stress test]. | □Coronary artery CT □Stress ECG □Stress scintigraphy □Stress echo □Stress MRI □FFR/Resting index □Others | |
| --- | --- | --- |
| Use of preoperative antiplatelet drugs | ○ Presence of | Presence or absence of antiplatelet drugs administered within 24 hours of PCI |
| Types of Preoperative Antiplatelet Drugs  ※ Enter "Yes" in [Use of preoperative antiplatelet drugs]. | ● Aspirin ● Clopidogrel ● Prasugrel ● Ticagrelor ● Other antiplatelet drugs | |
| Preoperative oral anticoagulant use | ○ Presence of | Presence or absence of anticoagulants administered within 24 hours of PCI |
| Types of preoperative oral anticoagulants  ※ Enter "Yes" in [Preoperative Oral Anticoagulants]. | ● Warfarin ● Dabigatran ● Rivaroxaban ● Apixaban ● Edoxaban ● Other antiplatelet drugs | |
| Pre operative Hb | [ ]g/d | Enter decimal digits between 1-24.0  The most recent value in the period up to 30 days before surgery |
| Pre operative Cr | [ ]mg/d  Dialysis patient | Enter decimal digits between 0.1 and 20.0  The most recent value in the period up to 30 days before surgery  Patients undergoing hemodialysis or peritoneal dialysis should be checked for "dialysis patients." |
| Number of lesions | ● 1 branch ● 2 branches ● 3 branches ● main trunk | Major coronary arteries with significant stenotic lesions ≧75% (measured 50%) according to AHA classification. For example, when 90% of lesions are found in the right coronary artery and 75% in the main trunk, they are written in "1 branch" and "main trunk".  The degree of stenosis should be defined according to the AHA criteria for all of the following. Multiple responses possible. |

**Surgery/Intervention Information Intraoperative Information**

| Date of PCI | Year Month Day | Completion is mandatory until the month. | |
| --- | --- | --- | --- |
| Age | Years | On the case registration system, it is automatically calculated from "patient birth date" and "date of PCI." | |
| Primary surgeon | Medical Registry Number | Supervising doctor | ○ ● Yes ● Unknown  ○ Medical Registry Number |
| Status of PCI | ○ Elective ● Emergency | Urgents: PCI elective not planned by the previous day: other planned PCIs | |
| Access site | ○ Femoral artery ● Radial artery ● Others | | |
| Door to balloon time  ※ With the diagnosis of PCI  Write if "Acute myocardial infarction (ST-segment elevation)" is selected. | ○ Presence ● min. Unknown | Time from hospital arrival to balloon inflation or thrombus aspiration for target lesion. Time is a half-square integral of three orders of magnitude. Enter "50" for "50 minutes." Range 1-500。 | |
| Fluoroscopy times | ○ Presence Minutes ○ Unknown | Time is a half-square integral of three orders of magnitude. Enter "050" for "50 minutes." Input range 001-500. | |
| Operative Time | Minutes | Time range from admission to discharge 10-999 Entry of an integer | |
| Amount of contrast medium used | [ ]ml | Range 1-1999 Integral Value Entry | |
| Use of assisted circulatory devices for PCI | ○ Presence ○ No | | |
| Device type  ※ Enter if "Yes" is selected in [Use of assisted circulation device for PCI] | □IABP  □PCPS  □Impella  □LVAD  ● Others (are expected to be approved devices in the future) | Timing of IABP  ※ Enter if "IABP" is selected on [Device Type] | 〇 Before and after surgery |
| Timing of PCPS  ※ Enter if "PCPS" is selected on [Device Type] | 〇 Before and after surgery | Timing of Impella  ※ Enter if "Impella" is selected on [Device Type] | 〇 Before and after surgery |
| Timing of LVAD  ※ Enter if "LVAD" is selected on [Device Type] | 〇 Before and after surgery | Other Timing  ※ Enter if "Other" is selected on [Device Type] | 〇 Before and after surgery |

| Site of the lesion | ● RCA (right corona)  ● Graft disease | Arterial) Variation ● Soybean | ● LM•LAD (left main coronary artery, anterior descending artery) and others | | | | □LCX | Circumflex branch of left coronary artery | |
| --- | --- | --- | --- | --- | --- | --- | --- | --- | --- |
| Lesion number | | Execution or not | | Lesion appearance  Enter if "Existence of practice" is "Available". | Success or failure  Successful lesions: Visual stenosis of target vessels ≦25% and no delay to peripheral vessels after PCI  A shadow [TIMI 3 flow]. | | | | Equipment used  If the device was not used due to difficulty in passing the wire, enter "Other". |
|  |  |  | |  |  |  |  | | ● Balloon ● Drug-eluting balloon |
|  |  |  | |  |  |  |  | | ● Metal Stent ● Drug-Eluting Stent |
|  |  | ○ Presence | | ○ New |  |  |  | | ● Bioabsorbable scaffolds |
|  | 1 | ○ No | | ○ Closing again | ○ Success | ○ Failure | ○ Unknown | | ● Rotator ● DCA ● Thrombus aspiration |
|  |  | ○ Unknown | | ○ Unknown |  |  |  | | ● Distal protection  ● Wire/device impermeable ● Others |
|  |  |  | |  |  |  |  | | Unknown |
|  |  |  | |  |  |  |  | | ● Balloon ● Drug-eluting balloon |
|  |  |  | |  |  |  |  | | ● Metal Stent ● Drug-Eluting Stent |
|  |  | ○ Presence | | ○ New |  |  |  | | ● Bioabsorbable scaffolds |
|  | 2 | ○ No | | ○ Closing again | ○ Success | ○ Failure | ○ Unknown | | ● Rotator ● DCA ● Thrombus aspiration |
|  |  | ○ Unknown | | ○ Unknown |  |  |  | | ● Distal protection  ● Wire/device impermeable ● Others |
|  |  |  | |  |  |  |  | | Unknown |
|  |  |  | |  |  |  |  | | ● Balloon ● Drug-eluting balloon |
|  |  |  | |  |  |  |  | | ● Metal Stent ● Drug-Eluting Stent |
| RCA (right coronary artery)  ※ "RCA (right coronary artery)" at "lesion site" | 3 | ○ Presence  ○ No | | ○ New  ○ Closing again | ○ Success | ○ Failure | ○ Unknown | | ● Bioabsorbable scaffolds  ● Rotator ● DCA ● Thrombus aspiration |
| If selected, enter the right. |  | ○ Unknown | | ○ Unknown |  |  |  | | ● Distal protection  ● Wire/device impermeable ● Others |
|  |  |  | |  |  |  |  | | Unknown |
|  |  |  | |  |  |  |  | | ● Balloon ● Drug-eluting balloon |
|  |  |  | |  |  |  |  | | ● Metal Stent ● Drug-Eluting Stent |
|  |  | ○ Presence | | ○ New |  |  |  | | ● Bioabsorbable scaffolds |
|  | 4PD | ○ No | | ○ Closing again | ○ Success | ○ Failure | ○ Unknown | | ● Rotator ● DCA ● Thrombus aspiration |
|  |  | ○ Unknown | | ○ Unknown |  |  |  | | ● Distal protection  ● Wire/device impermeable ● Others |
|  |  |  | |  |  |  |  | | Unknown |
|  |  |  | |  |  |  |  | | ● Balloon ● Drug-eluting balloon |
|  |  |  | |  |  |  |  | | ● Metal Stent ● Drug-Eluting Stent |
|  |  | ○ Presence | | ○ New |  |  |  | | ● Bioabsorbable scaffolds |
|  | 4AV | ○ No | | ○ Closing again | ○ Success | ○ Failure | ○ Unknown | | ● Rotator ● DCA ● Thrombus aspiration |
|  |  | ○ Unknown | | ○ Unknown |  |  |  | | ● Distal protection  ● Wire/device impermeable ● Others |
|  |  |  | |  |  |  |  | | Unknown |
| Lesion number | | Execution or not | | Lesion appearance  Enter if "Existence of practice" is "Available". | Success or failure  Successful Lesions: The degree of target vessel stenosis after PCI is visually less than 25% and does not delay to peripheral vessels [TIMI 3 flow]. | | | | Equipment used  If the device was not used due to difficulty in passing the wire, enter "Other". |
|  |  |  | |  |  |  |  | | ● Balloon ● Drug-eluting balloon |
|  |  |  | |  |  |  |  | | ● Metal Stent ● Drug-Eluting Stent |
|  |  | ○ Presence | | ○ New |  |  |  | | ● Bioabsorbable scaffolds |
|  | 5 | ○ No | | ○ Closing again | ○ Success | ○ Failure | ○ Unknown | | ● Rotator ● DCA ● Thrombus aspiration |
|  |  | ○ Unknown | | ○ Unknown |  |  |  | | ● Distal protection  ● Wire/device impermeable ● Others |
|  |  |  | |  |  |  |  | | Unknown |
| LM•LAD (left main coronary artery, anterior) |  |  | |  |  |  |  | | ● Balloon ● Drug-eluting balloon  ● Metal Stent ● Drug-Eluting Stent  ● Bioabsorbable scaffolds  ● Rotator ● DCA ● Thrombus aspiration  ● Distal protection  ● Wire/device impermeable ● Others  Unknown |
| Descending branch |  | ○ Presence | | ○ New |  |  |  | |  |
| ※ "LM•LAD" (left) at "lesion site" | 6 | ○ No | | ○ Closing again | ○ Success | ○ Failure | ○ Unknown | |  |
| To the main coronary artery and the anterior descending artery |  | ○ Unknown | | ○ Unknown |  |  |  | |  |
| If selected, enter the right. |  |  | |  |  |  |  | |  |
|  |  |  | |  |  |  |  | | ● Balloon ● Drug-eluting balloon |
|  |  |  | |  |  |  |  | | ● Metal Stent ● Drug-Eluting Stent |
|  |  | ○ Presence | | ○ New |  |  |  | | ● Bioabsorbable scaffolds |
|  | 7 | ○ No | | ○ Closing again | ○ Success | ○ Failure | ○ Unknown | | ● Rotator ● DCA ● Thrombus aspiration |
|  |  | ○ Unknown | | ○ Unknown |  |  |  | | ● Distal protection  ● Wire/device impermeable ● Others |
|  |  |  | |  |  |  |  | | Unknown |
|  |  |  | |  |  |  |  | | ● Balloon ● Drug-eluting balloon |
|  |  |  | |  |  |  |  | | ● Metal Stent ● Drug-Eluting Stent |
|  |  | ○ Presence | | ○ New |  |  |  | | ● Bioabsorbable scaffolds |
|  | 8 | ○ No | | ○ Closing again | ○ Success | ○ Failure | ○ Unknown | | ● Rotator ● DCA ● Thrombus aspiration |
|  |  | ○ Unknown | | ○ Unknown |  |  |  | | ● Distal protection  ● Wire/device impermeable ● Others |
|  |  |  | |  |  |  |  | | Unknown |

|  | 9 | ○ Presence  ○ No  ○ Unknown | ○ New  ○ Closing again  ○ Unknown | ○ Success | ○ Failure | ○ Unknown | ● Balloon ● Drug-eluting balloon  ● Metal Stent ● Drug-Eluting Stent  ● Bioabsorbable scaffolds  ● Rotator ● DCA ● Thrombus aspiration  ● Distal protection  ● Wire/device impermeable ● Others  Unknown |
| --- | --- | --- | --- | --- | --- | --- | --- |
|  | 10 | ○ Presence  ○ No  ○ Unknown | ○ New  ○ Closing again  ○ Unknown | ○ Success | ○ Failure | ○ Unknown | ● Balloon ● Drug-eluting balloon  ● Metal Stent ● Drug-Eluting Stent  ● Bioabsorbable scaffolds  ● Rotator ● DCA ● Thrombus aspiration  ● Distal protection  ● Wire/device impermeable ● Others  Unknown |
| Lesion number | | Execution or not | Lesion appearance  Enter if "Existence of practice" is "Available". | Success or failure  Successful Lesions: The degree of target vessel stenosis after PCI is visually less than 25% and does not delay to peripheral vessels [TIMI 3 flow]. | | | Equipment used  If the device was not used due to difficulty in passing the wire, enter "Other". |
| LCX (left circumflex coronary artery)  ※ Write the right when "LCX (left circumflex coronary artery)" is selected for "lesion site." | 11 | ○ Presence  ○ No  ○ Unknown | ○ New  ○ Closing again  ○ Unknown | ○ Success | ○ Failure | ○ Unknown | ● Balloon ● Drug-eluting balloon  ● Metal Stent ● Drug-Eluting Stent  ● Bioabsorbable scaffolds  ● Rotator ● DCA ● Thrombus aspiration  ● Distal protection  ● Wire/device impermeable ● Others  Unknown |
|  | HL/12 | ○ Presence  ○ No  ○ Unknown | ○ New  ○ Closing again  ○ Unknown | ○ Success | ○ Failure | ○ Unknown | ● Balloon ● Drug-eluting balloon  ● Metal Stent ● Drug-Eluting Stent  ● Bioabsorbable scaffolds  ● Rotator ● DCA ● Thrombus aspiration  ● Distal protection  ● Wire/device impermeable ● Others  Unknown |
|  | 13 | ○ Presence  ○ No  ○ Unknown | ○ New  ○ Closing again  ○ Unknown | ○ Success | ○ Failure | ○ Unknown | ● Balloon ● Drug-eluting balloon  ● Metal Stent ● Drug-Eluting Stent  ● Bioabsorbable scaffolds  ● Rotator ● DCA ● Thrombus aspiration  ● Distal protection  ● Wire/device impermeable ● Others  Unknown |
|  | 14 | ○ Presence  ○ No  ○ Unknown | ○ New  ○ Closing again  ○ Unknown | ○ Success | ○ Failure | ○ Unknown | ● Balloon ● Drug-eluting balloon  ● Metal Stent ● Drug-Eluting Stent  ● Bioabsorbable scaffolds  ● Rotator ● DCA ● Thrombus aspiration  ● Distal protection  ● Wire/device impermeable ● Others  Unknown |
|  | 15 | ○ Presence  ○ No  ○ Unknown | ○ New  ○ Closing again  ○ Unknown | ○ Success | ○ Failure | ○ Unknown | ● Balloon ● Drug-eluting balloon  ● Metal Stent ● Drug-Eluting Stent  ● Bioabsorbable scaffolds  ● Rotator ● DCA ● Thrombus aspiration  ● Distal protection  ● Wire/device impermeable ● Others  Unknown |

| Lesion number | | Execution or not | Lesion appearance  Enter if "Existence of practice" is "Available". | Success or failure  Successful Lesions: The degree of target vessel stenosis after PCI is visually less than 25% and does not delay to peripheral vessels [TIMI 3 flow]. | Equipment used  If the device was not used due to difficulty in passing the wire, enter "Other". |
| --- | --- | --- | --- | --- | --- |
| Graft lesion  ※ If "graft lesion" is selected in "lesion site", enter the right. | SVG  (LAD) | ○ Presence  ○ No  ○ Unknown | ○ New  ○ Closing again  ○ Unknown | ○ ● Success ● Failure ● Unknown | ● Balloon ● Drug-eluting balloon  ● Metal Stent ● Drug-Eluting Stent  ● Bioabsorbable scaffolds  ● Rotator ● DCA ● Thrombus aspiration  ● Distal protection  ● Wire/device impermeable ● Others  Unknown |
|  | SVG  (LCX) | ○ Presence  ○ No  ○ Unknown | ○ New  ○ Closing again  ○ Unknown | ○ ● Success ● Failure ● Unknown | ● Balloon ● Drug-eluting balloon  ● Metal Stent ● Drug-Eluting Stent  ● Bioabsorbable scaffolds  ● Rotator ● DCA ● Thrombus aspiration  ● Distal protection  ● Wire/device impermeable ● Others  Unknown |
|  | SVG  (RCA) | ○ Presence  ○ No  ○ Unknown | ○ New  ○ Closing again  ○ Unknown | ○ ● Success ● Failure ● Unknown | ● Balloon ● Drug-eluting balloon  ● Metal Stent ● Drug-Eluting Stent  ● Bioabsorbable scaffolds  ● Rotator ● DCA ● Thrombus aspiration  ● Distal protection  ● Wire/device impermeable ● Others  Unknown |
|  | AG  (LAD) | ○ Presence  ○ No  ○ Unknown | ○ New  ○ Closing again  ○ Unknown | ○ ● Success ● Failure ● Unknown | ● Balloon ● Drug-eluting balloon  ● Metal Stent ● Drug-Eluting Stent  ● Bioabsorbable scaffolds  ● Rotator ● DCA ● Thrombus aspiration  ● Distal protection  ● Wire/device impermeable ● Others  Unknown |
|  | AG  (LCX) | ○ Presence  ○ No  ○ Unknown | ○ New  ○ Closing again  ○ Unknown | ○ ● Success ● Failure ● Unknown | ● Balloon ● Drug-eluting balloon  ● Metal Stent ● Drug-Eluting Stent  ● Bioabsorbable scaffolds  ● Rotator ● DCA ● Thrombus aspiration  ● Distal protection  ● Wire/device impermeable ● Others  Unknown |
|  | AG  (RCA) | ○ Presence  ○ No  ○ Unknown | ○ New  ○ Closing again  ○ Unknown | ○ ● Success ● Failure ● Unknown | ● Balloon ● Drug-eluting balloon  ● Metal Stent ● Drug-Eluting Stent  ● Bioabsorbable scaffolds  ● Rotator ● DCA ● Thrombus aspiration  ● Distal protection  ● Wire/device impermeable ● Others  Unknown |
| Lesion number | | Execution or not | Lesion appearance  Enter if "Existence of practice" is "Available". | Success or failure  Successful Lesions: The degree of target vessel stenosis after PCI is visually less than 25% and does not delay to peripheral vessels [TIMI 3 flow]. | Equipment used  If the device was not used due to difficulty in passing the wire, enter "Other". |
| Other  ※ If "Other" is selected in "Location of lesion", enter the right. |  | ○ Presence  ○ No  ○ Unknown | ○ New  ○ Closing again  ○ Unknown | ○ ● Success ● Failure ● Unknown | ● Balloon ● Drug-eluting balloon  ● Metal Stent ● Drug-Eluting Stent  ● Bioabsorbable scaffolds  ● Rotator ● DCA ● Thrombus aspiration  ● Distal protection  ● Wire/device impermeable ● Others  Unknown |

**Surgery and Intervention Information Postoperative Information**

| Complication of the procedure | ○ Presence ○ No  If "yes", enter the following:  ● Myocardial infarction due to PCI procedure  Cardiac tamponade ● Heart failure/shock requiring circulatory support or inotropic drugs  ● Stent thrombosis (in-hospital onset) Emergency surgery  ● Hemorrhagic complications requiring blood transfusion (access site)  ● Bleeding complications requiring transfusion (non-access site)  Other | See Complications of the procedure on the cover of the J-PCI. Multiple responses possible. |
| --- | --- | --- |

| Hospital mortality | 〇 Presence 〇 No | See "In-hospital deaths" on the cover of the J-PCI. |
| --- | --- | --- |
| In-hospital death date | Indication when [In-hospital death] is "Yes"  Western calendar Year Month Day | |
| Causa mortis | Indication when [In-hospital death] is "Yes"  Cardiac death  ● Procedure-related death (optional if cardiac death)  ● Other cardiac deaths (if cardiac death is acceptable)  ● Non-cardiac death | See "In-hospital deaths" on the cover of the J-PCI. |
